# Bis(4-hydroxyphenyl) Sulfone Regulates Sperm Motility, Mitochondrial Activity, and Nitric Oxide Production in Boar Semen

**DOI:** 10.3390/ani16193093

**Published:** 2026-10-01

**Authors:** Seunghyung Lee, Sang-Hee Lee

**Affiliations:** 1 College of Animal Life Sciences, Kangwon National University, Chuncheon 24341, Republic of Korea; s.lee@kangwon.ac.kr; 2 School of Information and Communication Technology, University of Tasmania, Hobart, TAS 7005, Australia

**Keywords:** bis(4-hydroxyphenyl) sulfone, boar, sperm

## Abstract

BPS is a substitute for bisphenol A, an endocrine disruptor. Our study aimed to evaluate the effects of BPS on sperm viability and motility, acrosomal membrane damage, mitochondrial activity, and NO production in boar semen. 0, 40.0, 80.0, and 100.0 μM BPS were treated in the boar semen. In the results, sperm viability did not change across any treatments. However, sperm motility was significantly decreased in the 100.0 μM BPS-treated groups (78.56%, *p* < 0.05) and progressive motility of sperm (60.24%, *p* < 0.05). Acrosomal membrane damage was not changed in the BPS-treated groups, but mitochondrial activity (58.85%, *p* < 0.05) of sperm was decreased in the 100.0 μM BPS-treated groups. The NO in the 100.0 μM BPS-treated sperm groups was increased (*p* < 0.05). Based on the results, we suggest that BPS elevates NO production, and the increased NO damages mitochondrial activity. Thus, BPS may directly regulate sperm motility, such as through oxidative stress.

## Simple Summary

Bis(4-hydroxyphenyl) sulfone (BPS) is an endocrine disruptor, similar to bisphenol A. BPS can influence hormone-regulated processes and cellular functions related to reproduction. Thus, we evaluated sperm viability, motility, acrosomal membrane damage, mitochondrial activity, and nitric oxide in boar semen. In the results, BPS can regulate the motility of sperm and mitochondrial activity in boar semen. Also, BPS stimulated the level of nitric oxide (NO) production. Our results suggest that BPS may cause a loss of sperm function in boar semen.

## 1. Introduction

Bisphenol A, a synthetic chemical widely used in the manufacture of plastics and epoxy resins, has been extensively studied for its reproductive toxicity [1,2]. In response to regulatory restrictions and consumer concerns regarding bisphenol A exposure, structurally related compounds such as Bis(4-hydroxyphenyl) sulfone (BPS) have increasingly been adopted as alternative industrial chemicals. BPS is now commonly detected in thermal paper receipts, food packaging materials, personal care products, and environmental factors [3,4,5]. In particular, BPS has been detected in thermal receipt paper at concentrations ranging from 0.0000138 to 22.0 mg/g paper [6]. Although initially introduced as a safer substitute for bisphenol A, accumulating research suggests that BPS has endocrine-disrupting properties comparable to bisphenol A and may adversely affect reproductive function.

From a practical point of view, boars may be exposed to BPS through feed and materials used to store or handle feed. Previous studies have detected BPS and other bisphenol compounds in animal feed and plastic feed packaging, suggesting that feed may be one route of exposure [7,8]. BPS taken orally can also be absorbed in pigs, indicating that contaminated feed may lead to exposure in the body [9]. In addition, boar semen is commonly stored and transported in plastic bags or containers, and some studies have shown that chemicals released from these materials can enter extended semen and affect sperm function [10,11]. These findings suggest that exposure through feed and contact of semen with plastic materials should be considered as possible routes of BPS exposure in boars and boar semen.

Studies in animals have demonstrated that BPS exposure can negatively affect multiple aspects of male reproductive physiology [2]. Some research groups reported that the effects include reductions in sperm count and motility, increased oxidative stress, disruption of testicular steroidogenesis, altered reproductive hormone concentrations, and increased germ-cell apoptosis [2,3,4]. These adverse outcomes have been observed in rodents exposed during developmental and adult life stages, suggesting that BPS may interfere with reproductive processes throughout the lifespan. Furthermore, mechanistic investigations indicate that oxidative stress, mitochondrial dysfunction, and endocrine disruption are among the primary pathways through which BPS exerts reproductive toxicity [3,4]. In pigs, in vitro studies have also shown that BPS can affect meiotic maturation, spindle formation, cumulus cell function, and mitochondrial distribution in oocytes and cumulus-oocyte complexes [12,13]. In human studies, although more limited, they also raise concerns regarding the reproductive effects of BPS. In a cohort of men attending a fertility clinic, urinary BPS concentrations were associated with poorer semen quality parameters, including lower sperm concentration, total sperm count, and sperm motility, particularly among overweight and obese individuals [14,15]. Similarly, measurements of BPS in seminal plasma have revealed negative relationships between BPS exposure and sperm concentration, suggesting that environmental exposure to this compound may contribute to impaired male fertility [3]. In addition to epidemiological observations, in vitro studies using human spermatozoa have demonstrated that exposure to BPS can alter sperm functional characteristics [3]. Although the underlying mechanisms are not yet fully understood, available evidence suggests involvement of oxidative stress pathways, membrane destabilization, and disturbances in cellular signaling processes that regulate sperm motility and fertilization competence [16]. These findings support concerns that BPS may directly impair sperm function even at environmentally relevant exposure levels.

Sperm function encompasses several critical processes, including motility, capacitation, acrosomal reaction, mitochondrial activity, membrane integrity, and maintenance of DNA stability. These functions are highly sensitive to alterations in hormonal balance and oxidative status. Because mature spermatozoa possess limited antioxidant defenses and DNA repair mechanisms, they are particularly vulnerable to environmental toxicants that induce oxidative stress [2,4]. Given the widespread use of BPS and increasing human and animal exposure, understanding its potential effects on male reproductive health has become an important area of research. Current evidence from experimental animal models and limited human studies suggests that BPS can adversely affect sperm quality and reproductive function. However, important knowledge gaps remain regarding dose–response relationships, exposure thresholds, the molecular mechanisms underlying its reproductive toxicity, and the long-term consequences of chronic exposure [17,18]. Further investigation is therefore necessary to clarify the risks associated with BPS exposure and to determine whether this BPA substitute represents a safer alternative or merely a replacement with similar reproductive hazards. There is no study on the relationship between BPS and nitric oxide in boar sperm. Thus, we conducted a study on the effects of BPS on sperm viability, motility, acrosomal membrane damage, mitochondrial membrane integrity, and nitric oxide production in boar semen.

## 2. Materials and Methods

### 2.1. Chemicals and Experimental Design

Bis(4-hydroxyphenyl) sulfone (BPS) and the other chemicals used in this study were obtained from Sigma-Aldrich (St. Louis, MO, USA), unless otherwise specified. The effects of BPS exposure on boar sperm function were examined by assessing sperm motility, viability, acrosomal membrane damage, mitochondrial activity, and nitric oxide production.

### 2.2. Preparation and Treatment of Boar Semen

Semen samples were obtained from ten Duroc boars at the Artificial Insemination Center (Wonju, Republic of Korea) and initially diluted with Modena extender [19]. On each experimental day, semen samples from two different boars were available, and one sample was randomly selected for the experiment. This procedure was repeated on five separate occasions, resulting in five independent ejaculates from five boars. Following collection, the diluted semen was transported to the laboratory within 1 h. Upon arrival, we adjusted the samples to a final concentration of 1.0 × 10^7^ sperm/mL using Modena extender (glucose 30.0 g/L, EDTA 2.25 g/L, sodium citrate 6.90 g/L, sodium bicarbonate 1.00 g/L, Tris 5.00 g/L, citric acid 2.50 g/L, cysteine 0.05 g/L, gentamicin sulfate 0.30 g/L). Each ejaculate was divided into four aliquots and treated with 0, 40.0, 80.0, or 100.0 μM BPS. BPS was dissolved in dimethyl sulfoxide, and the final dimethyl sulfoxide concentration was maintained at 0.3% in all groups, including the control. All sperm were stored and incubated at 17 °C for 3 h. We used the treated samples to evaluate sperm motility, viability, acrosomal membrane damage, mitochondrial activity, and nitric oxide production. All experimental procedures were conducted in accordance with institutional guidelines and were approved by the Institutional Animal Care and Use Committee of Kangwon National University, Chuncheon, The Republic of Korea (Approval No. KW-250716-3).

### 2.3. Analysis of Sperm Motility

Sperm motility was assessed using a computer-assisted sperm analysis system (CASA; Sperm Class Analyzer^®^ v6.6, Microptic, Barcelona, Spain). A 10 μL aliquot of each diluted sperm sample was placed in a 20 μm chamber slide (Leja, Nieuw-Vennep, The Netherlands) maintained at 38.5 °C on a heated stage. At least five randomly selected microscopic fields were examined using a phase-contrast microscope equipped with a 10× objective lens (BX51, Olympus, Tokyo, Japan), and approximately 200 sperm were evaluated per sample. Video images were recorded at 30 frames/s, and 30 consecutive frames were analyzed for each field. Sperm heads were detected using an area range of 10–80 μm^2^. The recorded motility parameters included total motility (TM, %), progressive motility (PM, %), average path velocity (VAP, μm/s), straight-line velocity (VSL, μm/s), and curvilinear velocity (VCL, μm/s). The default analysis settings provided by the SCA software v6.6 for boar sperm were used for the calculation and classification of these motility parameters, without manual modification.

### 2.4. Assessment of Sperm Viability

Sperm viability was determined using a LIVE/DEAD sperm viability kit (L-7011, Invitrogen, New York, NY, USA). Staining and evaluation were performed according to the procedure described in a previous study [20].

### 2.5. Evaluation of Acrosomal Membrane Damage and Mitochondrial Activity

Acrosomal membrane damage and mitochondrial activity were evaluated by fluorescent staining followed by flow cytometric analysis, with modifications based on a previously described procedure [21]. For the assessment of acrosomal membrane damage, sperm samples were stained with 2.0 μM fluorescein isothiocyanate-conjugated peanut agglutinin from Arachis hypogaea (FITC-PNA; Sigma-Aldrich) and 2.0 μM propidium iodide (PI; Sigma-Aldrich). The stained samples were incubated at 38 °C for 5 min and subsequently centrifuged at 410× *g* for 5 min. After removal of the supernatant, the sperm pellets were resuspended in 1.0 mL phosphate-buffered saline (PBS). Mitochondrial activity was assessed using Rhodamine 123 and PI double staining. Sperm samples were incubated with 2.4 μM Rhodamine 123 and 2.0 μM PI at 38 °C for 10 min. Following incubation, the samples were centrifuged at 410× *g* for 5 min, the supernatant was removed, and the sperm pellets were resuspended in 1.0 mL PBS. For each sample, 10,000 sperm events were acquired using an Attune CytPix flow cytometer (Thermo Fisher Scientific, Waltham, MA, USA) equipped with a 488 nm laser. Sperm populations were identified using forward scatter and side scatter characteristics, and fluorescence signals were evaluated using the FL1 and FL2 channels. The acquired data were processed using Attune software 6.2 (Thermo Fisher Scientific). Sperm exhibiting FITC-PNA-positive fluorescence, irrespective of PI staining status, were classified as having acrosomal membrane damage. Accordingly, the FITC-PNA^+^/PI^−^ and FITC-PNA^+^/PI^+^ populations were included in the damaged-acrosome population. Sperm showing Rhodamine 123-positive and PI-negative fluorescence were classified as viable sperm with intact mitochondrial activity.

### 2.6. Nitric Oxide

Intracellular nitric oxide production was evaluated using 4,5-diaminofluorescein diacetate (DAF-2 DA; Sigma-Aldrich). Nitric oxide levels in BPS-treated boar sperm samples were measured according to previously described procedures [19,22]. Briefly, we washed the sperm in PBS for 10 min at 400× *g*, then DAF-2 DA was added to the suspension to a final concentration of 10.0 μM. Then, the sperm were incubated for an hour at 37.0 °C (in a dark room). Finally, the sperm were centrifuged at 400× *g* (5 min), and we measured the sperm samples for NO using flow cytometry.

### 2.7. Statistical Analysis

The normality of the data was assessed using the Shapiro–Wilk test (SAS 9.4 Institute, Cary, NC, USA). Differences among BPS treatment groups were analyzed using one-way ANOVA followed by Bonferroni’s multiple comparison test, with each boar treated as the paired experimental unit. Data are presented as the mean ± standard error of the mean (SEM). A *p*-value < 0.05 was considered statistically significant.

## 3. Results

### 3.1. Effect of BPS on Sperm Viability and Motility in Boar Semen

**Figure 1:**
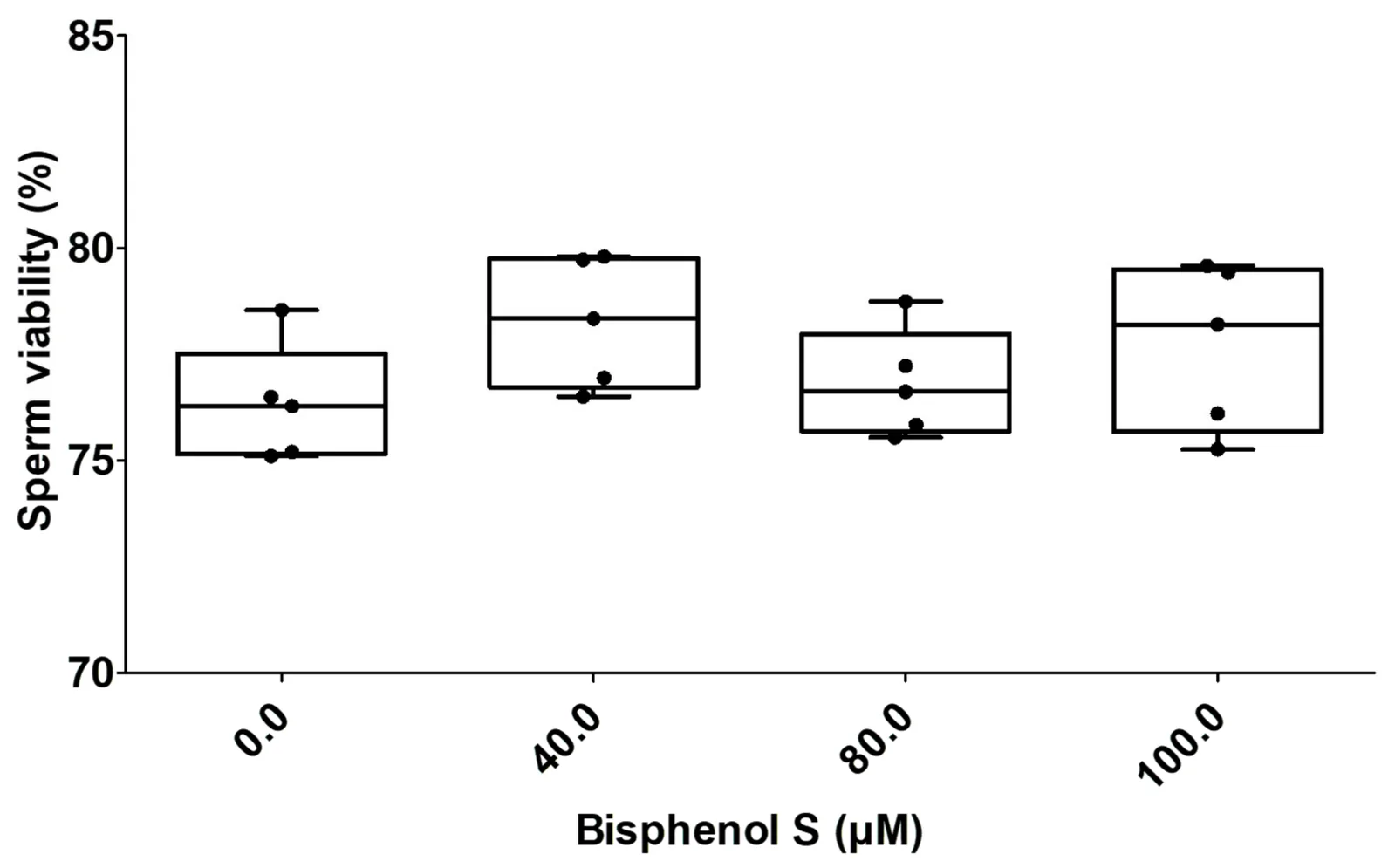
Dose-dependent effect of BPS on the viability of sperm in boar semen. Bars represent means ± standard error (*p* = 0.345).

**Figure 2:**
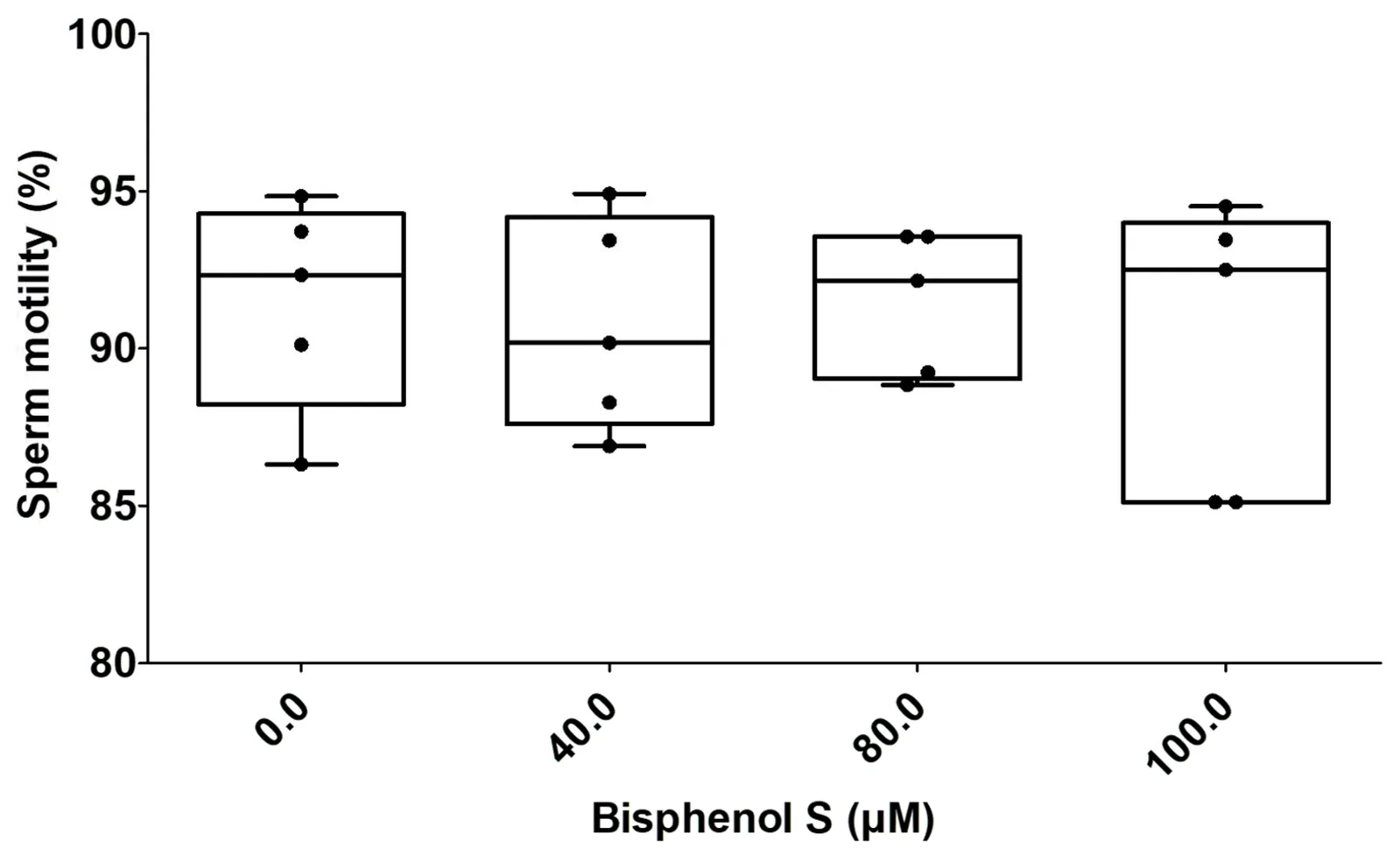
Dose-dependent effect of BPS on the motility of sperm in boar semen. Bars represent means ± standard error (*p* = 0.908).

Sperm viability was not changed in the 0, 40.0, 80.0, and 100.0 μM BPS-treated groups (Figure 1). As shown in Figure 2, we measured sperm motility in the BPS-treated semen groups (0, 40.0, 80.0, and 100.0 μM) immediately. The motility of sperm in all treated groups did not change. However, sperm motility (78.56%) was significantly decreased in the 100.0 μM BPS-treated group (*p* < 0.05, Table 1). Progressive motility (60.24%) of sperm was also decreased in the 100.0 μM BPS-treated group (*p* < 0.05, Table 1). But the VAP, VSL, and VCL of sperm in the 0, 40.0, 80.0, and 100.0 μM BPS-treated groups did not change (Table 1).

### 3.2. Effect of BPS on Acrosomal Membrane Damage and Mitochondrial Activity in Boar Sperm

**Figure 3:**
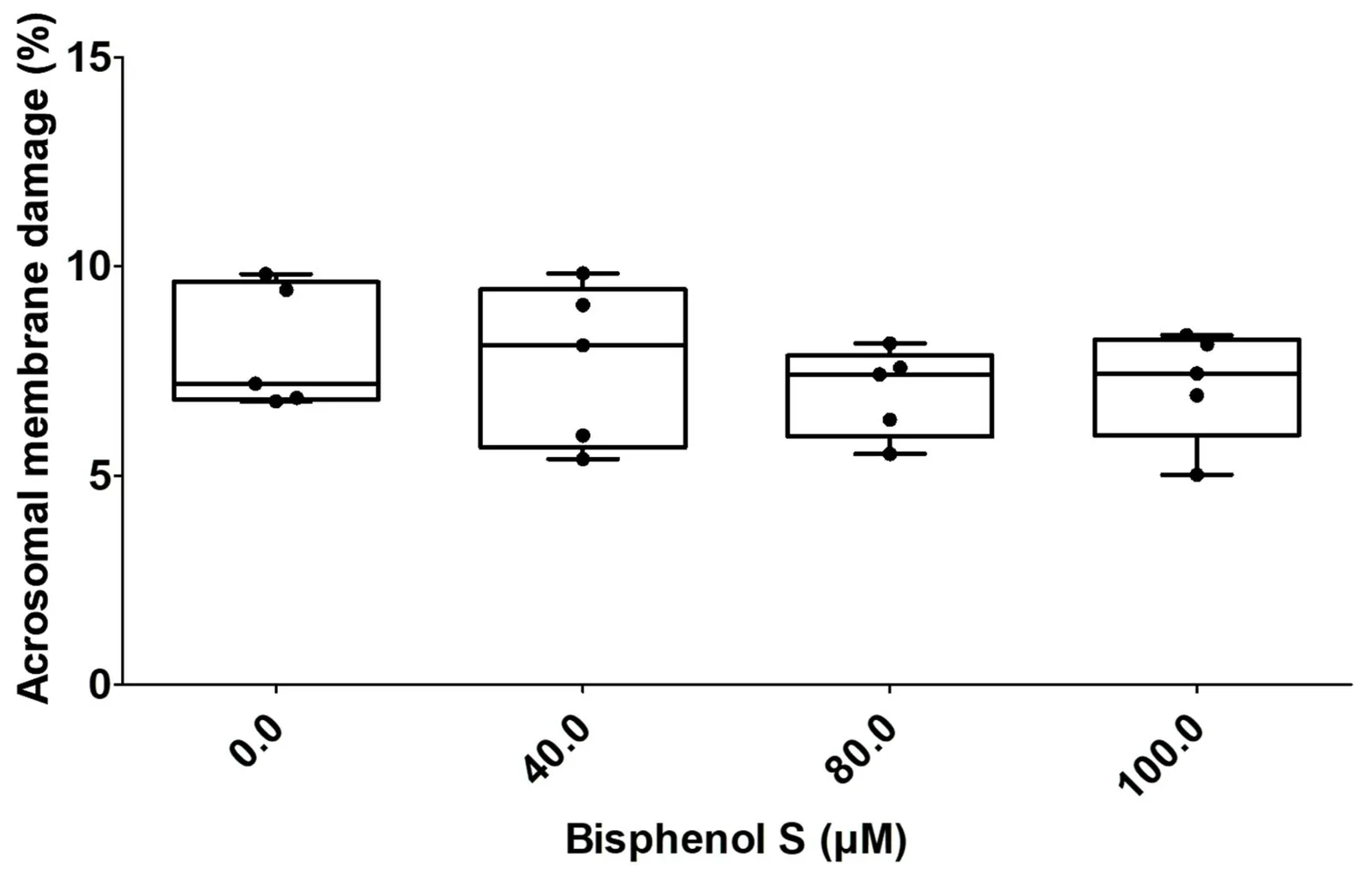
Effect of BPS on the damage of the acrosomal membrane in boar semen. Sperm were treated with 0.0, 40.0, 80.0, and 100.0 μM BPS. Bars represent means ± standard error (*p* = 0.772).

**Figure 4:**
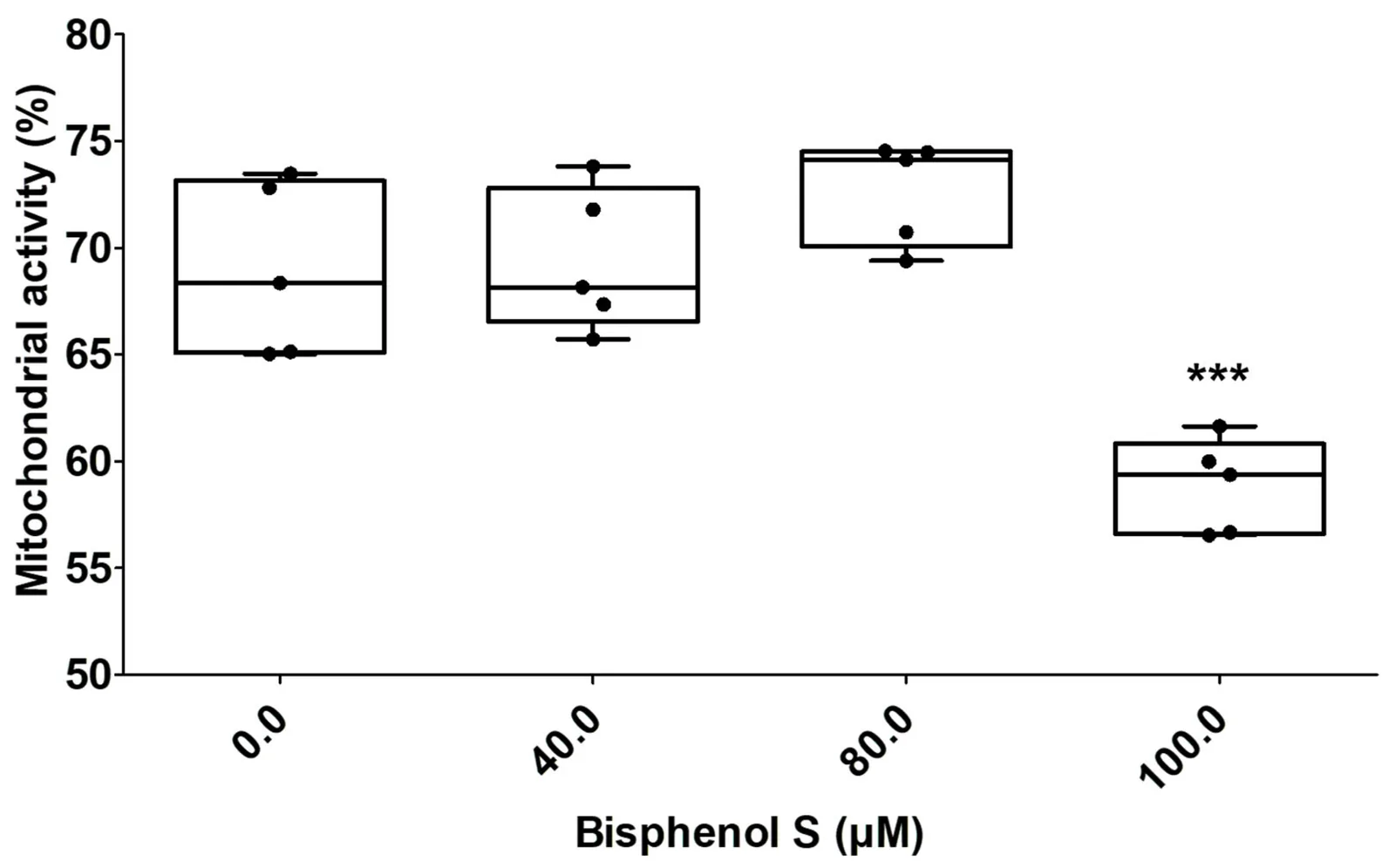
Effect of BPS on mitochondrial activity in boar semen. Sperm were treated with 0.0, 40.0, 80.0, and 100.0 μM BPS. Bars represent means ± standard error. Asterisks indicate significant differences (***, *p* < 0.001).

As shown in Figure 3 and Figure 4, acrosomal membrane damage and mitochondrial activity in the BPS-treated (0, 40.0, 80.0, and 100.0 μM) sperm groups were evaluated. The acrosomal membrane damage did not change between the BPS-treated sperm groups (Figure 3). Mitochondrial activity was significantly decreased in the 100.0 μM BPS-treated sperm group (58.85%, *p* < 0.05) compared to the other groups (Figure 4).

### 3.3. Effect of BPS on the Production of Nitric Oxide in Boar Sperm

**Figure 5:**
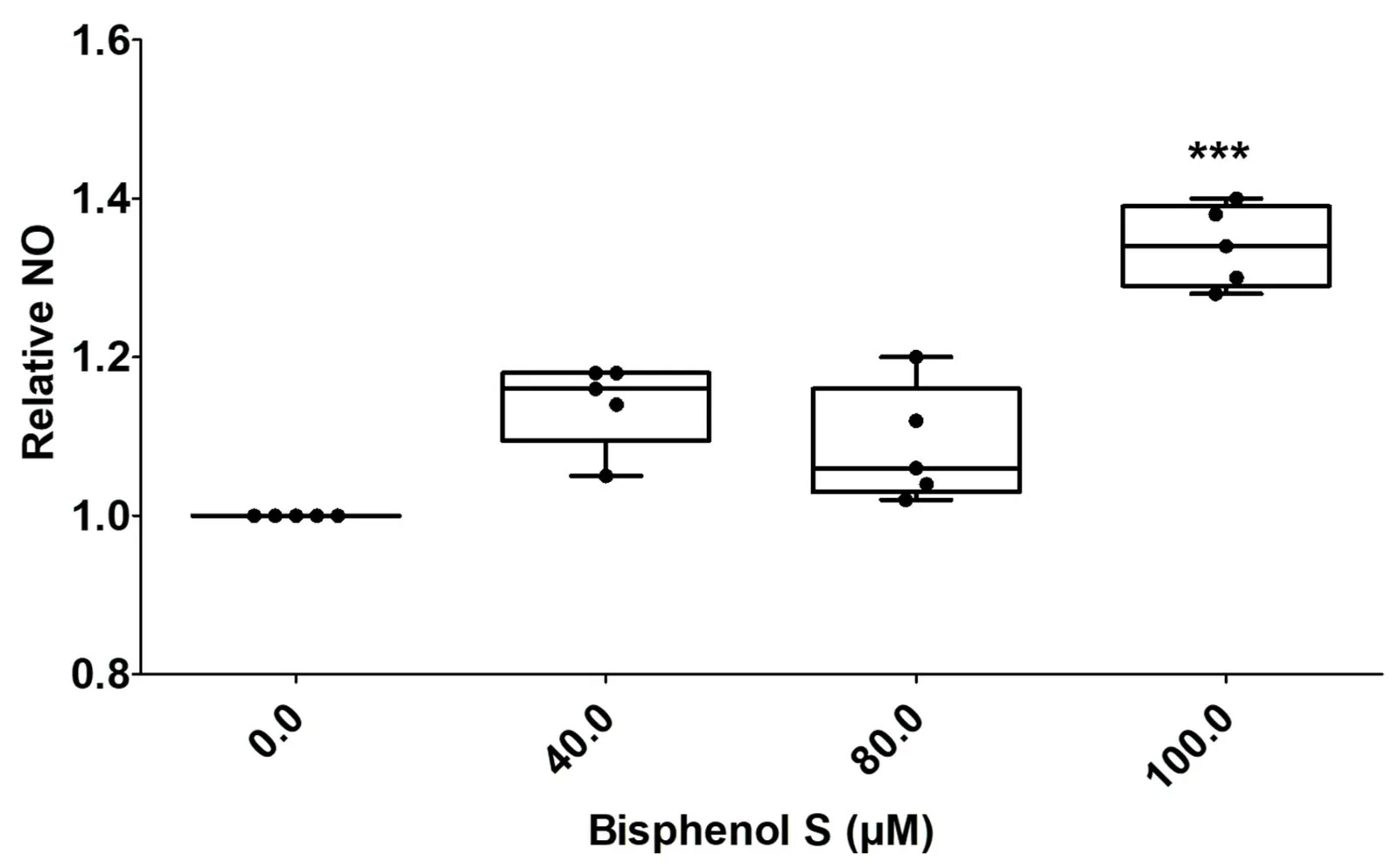
Effect of BPS on nitric oxide in boar semen. Sperm were treated with 0.0, 40.0, 80.0, and 100.0 μM BPS. Bars represent means ± standard error. Asterisks indicate significant differences (***, *p* < 0.001).

As shown in Figure 5, nitric oxide levels in boar sperm were stimulated by BPS. The production of nitric oxide in the 100.0 μM BPS-treated group was significantly increased (*p* < 0.05). But the 40 and 80 μM BPS-treated groups did not change. Although it was not significantly increased at the low concentration of BPS, nitric oxide was elevated in the 40 and 80 μM BPS-treated sperm compared to the untreated group.

## 4. Discussion

The findings of the present study provide additional evidence that BPS, a widely used substitute for bisphenol A, may adversely influence sperm function in animals. Although BPS has been introduced as a replacement compound with the intention of reducing health concerns associated with BPA exposure, increasing toxicological evidence suggests that it retains endocrine-disrupting activity and may compromise male reproductive performance. The observed alterations in total sperm motility, progressive motility, mitochondrial activity, and nitric oxide production suggest that BPS exposure may influence several physiological processes related to sperm function.

Sperm motility is a fundamental determinant of male fertility because successful migration through the female reproductive tract requires adequate energy production and coordinated flagellar movement [23]. In the present study, the reduction in motile sperm following BPS exposure may be linked to disturbances in cellular redox balance. Some research groups have demonstrated that BPS can enhance the generation of reactive oxygen species in reproductive tissues while simultaneously weakening endogenous antioxidant defense systems [1,23,24]. Because spermatozoa possess limited cytoplasmic antioxidant capacity, they are highly vulnerable to oxidative damage. Excessive reactive oxygen species may alter membrane fluidity, disrupt ion channel activity, and impair axonemal proteins responsible for flagellar motion, ultimately reducing the proportion of motile sperm cells.

Moreover, a similar explanation may account for the decline in progressive motility. Progressive movement requires not only flagellar beating but also sufficient ATP availability to sustain directional propulsion [23,25,26]. Experimental evidence indicates that endocrine-disrupting chemicals can interfere with mitochondrial bioenergetics, thereby limiting energy production required for efficient sperm transport [26,27]. Reduced progressive motility observed after BPS exposure may therefore reflect compromised mitochondrial performance rather than solely structural damage to the sperm tail. Since progressive motility is often more closely associated with fertilizing capacity than total motility, such alterations may have significant implications for reproductive success.

A previous study using boar sperm also reported that 100 μM BPS reduced both total and progressive motility after 3 h of exposure, whereas a lower concentration did not affect these parameters [28]. These findings are consistent with the reductions in total and progressive motility observed in the present study. However, the previous study also reported changes in motility, whereas VAP, VSL, and VCL were not significantly changed in our study. Differences in incubation temperature, medium composition, sperm preparation, and semen pooling may have contributed to these different responses. Similar to the previous study, BPS did not significantly reduce sperm viability in our experiment [28]. This suggests that the decrease in sperm motility observed after BPS exposure was not simply due to decreased sperm viability. In addition, acrosomal membrane damage was not changed in the present study.

In our results, the decrease in mitochondrial activity suggests that mitochondrial function may be involved in the response of boar sperm to BPS exposure. Mitochondria located within the sperm mid-piece are essential for ATP synthesis and cellular survival. Maintenance of mitochondrial membrane potential is necessary for efficient oxidative phosphorylation and regulation of intracellular signaling pathways [16,25,26]. Several studies have reported that BPS exposure disrupts mitochondrial function, resulting in membrane depolarization, increased electron leakage, and excessive reactive oxygen species generation [23,28]. Previous studies suggest that mitochondrial dysfunction may increase oxidative stress and reduce cellular energy production, which could be related to reduced sperm function. These mechanisms may be relevant to the concurrent changes in mitochondrial activity and motility observed in the present study.

Interestingly, a previous study in boar sperm reported that BPS did not significantly alter mitochondrial membrane potential at 3 and 20 h, although mitochondrial reactive species increased after 20 h of exposure [28]. In contrast, our Rhodamine 123-based analysis showed reduced mitochondrial activity at 100 μM BPS after 3 h. Because mitochondrial membrane potential, mitochondrial reactive species, and Rhodamine 123-based mitochondrial activity represent different aspects of mitochondrial function, these findings are not directly equivalent. Also, BPS-related mitochondrial changes have also been reported in porcine reproductive cells such as oocytes, including alterations in mitochondrial distribution and mitochondrial-associated proteins [12,13]. These findings further suggest that mitochondria may be one of the cellular targets affected by BPS in porcine reproductive cells.

Nitric oxide was another parameter that changed following BPS exposure in the present study. Under physiological conditions, NO functions as a signaling molecule involved in the regulation of sperm motility, capacitation, acrosomal reaction, and mitochondrial activity [29,30]. However, the biological effects of NO are concentration-dependent. Moderate levels are generally beneficial for sperm function, whereas excessive production can contribute to nitrosative stress and cellular injury [31]. Exposure to endocrine-disrupting compounds has been associated with altered nitric oxide synthase activity and dysregulated NO generation in reproductive tissues [32]. Consequently, changes in NO levels following BPS exposure may reflect a disturbance of redox signaling pathways that are necessary for normal sperm physiology. Elevated NO concentrations may react with superoxide anions to form peroxynitrite, a highly reactive oxidant capable of damaging lipids, proteins, and nucleic acids. Conversely, insufficient NO production may impair signaling mechanisms required for optimal sperm movement and fertilization competence. To our knowledge, the previous study examining BPS in boar sperm did not evaluate NO production [28]. Therefore, the increase in NO observed in the present study provides additional information on the cellular response of boar sperm to BPS.

The relationship between mitochondrial function and nitric oxide signaling may also be relevant to the effects of BPS on sperm. Mitochondria represents both a source and a target of reactive nitrogen species, creating a close functional connection between energy metabolism and redox homeostasis [33,34]. Disturbances in NO production may compromise mitochondrial respiration, while mitochondrial injury may further enhance oxidative and nitrosative stress. In the present study, the decrease in mitochondrial activity and increase in NO production occurred simultaneously after BPS exposure. Although these changes may be related, the present study did not directly determine a causal relationship between NO production and mitochondrial activity. Because sperm cells possess limited cytoplasmic antioxidant reserves, they are particularly susceptible to oxidative injury. Changes in mitochondrial activity may therefore be associated with the reduced motility observed following BPS treatment.

## 5. Conclusions

Taken together, we cautiously propose that our results show that BPS exposure was associated with reduced sperm motility, progressive motility, and mitochondrial activity, together with increased NO production. These simultaneous changes suggest that mitochondrial function and NO-related signaling may be involved in the effects of BPS on boar sperm; however, the present study does not establish direct causal relationships among NO production, oxidative stress, mitochondrial activity, and sperm motility. Given the widespread use of BPS-containing products, continued research is required to determine the long-term reproductive consequences of exposure and to evaluate whether BPS represents a genuinely safer alternative to BPA.

## Figures and Tables

**Table 1:** Comparison of BPS-treated concentrations on motility in boar sperm. Sperm motility was analyzed using Computer-assisted sperm analysis (CASA).

BPS (μM)	TM (%)	PM (%)	VAP (μm/s)	VSL (μm/s)	VCL (μm/s)
0.0	83.85 ± 2.97	72.94 ± 2.48	75.08 ± 1.29	69.38 ± 1.95	138.90 ± 4.33
40.0	87.42 ± 1.06	68.93 ± 1.12	73.00 ± 1.35	72.25 ± 0.67	148.87 ± 5.21
80.0	86.62 ± 1.36	68.82 ± 1.29	76.14 ± 1.68	69.92 ± 1.11	141.48 ± 2.87
100.0	78.56 ± 1.20 *	60.24 ± 1.05 ***	74.20 ± 1.38	69.34 ± 1.81	146.85 ± 2.32
*p*-value	0.029	0.001	0.384	0.577	0.092

* Values in the same row with different superscripts are significantly different (*, *p* < 0.05, ***, *p* < 0.001), mean ± SEM. Total motility, TM; Progressive motility, PM; Velocity average path, VAP; Velocity straight line, VSL; Velocity curvilinear, VCL.

## Data Availability

The original contributions presented in this study are included in the article. Further inquiries can be directed to the corresponding author.
